# P2X Receptor-Dependent Modulation of Mast Cell and Glial Cell Activities in Neuroinflammation

**DOI:** 10.3390/cells10092282

**Published:** 2021-09-02

**Authors:** Barbora Salcman, Karen Affleck, Silvia Bulfone-Paus

**Affiliations:** 1 Lydia Becker Institute of Immunology and Inflammation, Manchester Collaborative Centre for Inflammation Research, University of Manchester, Manchester M13 9NT, UK; barbora.salcman@postgrad.manchester.ac.uk; 2 GlaxoSmithKline, Immunology Research Unit, Stevenage SG1 2NY, UK; karen.x.affleck@gsk.com

**Keywords:** mast cells, microglia, astrocytes, oligodendrocytes, P2X receptors

## Abstract

Localisation of mast cells (MCs) at the abluminal side of blood vessels in the brain favours their interaction with glial cells, neurons, and endothelial cells, resulting in the activation of these cells and the release of pro-inflammatory mediators. In turn, stimulation of glial cells, such as microglia, astrocytes, and oligodendrocytes may result in the modulation of MC activities. MCs, microglia, astrocytes, and oligodendrocytes all express P2X receptors (P2XRs) family members that are selectively engaged by ATP. As increased concentrations of extracellular adenosine 5′-triphosphate (ATP) are present in the brain in neuropathological conditions, P2XR activation in MCs and glial cells contributes to the control of their communication and amplification of the inflammatory response. In this review we discuss P2XR-mediated MC activation, its bi-directional effect on microglia, astrocytes and oligodendrocytes and role in neuroinflammation.

## 1. Introduction

Mast cells (MCs) are immune cells that form part of the innate branch of the immune system. Since MCs express a broad spectrum of high affinity receptors (e.g., high-affinity IgE receptor (FcɛRI), Fc-gamma receptor (FcγR), complement receptors and purinergic receptors), they rapidly respond to a variety of environmental and immune stimuli, resulting in the release of pre-formed mediators such as histamine and then later the production of newly synthesized cytokines, chemokines, growth factors, proteases, and lipid mediators [1,2,3].

MCs are of dual hematopoietic origin. In mice, a first wave of MCs originates during embryonic development from yolk-sac progenitors followed by a second wave of bone marrow-derived MCs in adulthood. This duality in the origin of MCs may influence the cell phenotype and function in various tissues [4,5]. After leaving the bone marrow, MC committed progenitors circulate in the bloodstream and mature in peripheral tissues under the influence of a cocktail of growth factors that include the stem cell factor [6,7].

Human MCs are found in low numbers in the hypothalamus, leptomeninges, area postrema, and the dura matter of the spinal cord [8]. Nearly 97% of all MCs found in the brain are positioned in the abluminal side of the brain blood vessels, which allows them to communicate with neurons, glial cells (such as astrocytes and microglia) and endothelial cells [8,9]. In the human brain, MC density was found to be less than <5 MCs in 5 μm thick tissue sections in meninges and perivascular area. However, during viral, bacterial and parasitic infections, MC numbers were observed to be higher, at around 11–20 cells per 5 μm thick tissue section in meninges and around 5–20 in perivascular area [10]. In the healthy brain the predominant MC phenotype is tryptase^+^chymase^+^ and their overall numbers can be affected by trauma and/or stress, whilst their activation could potentially influence social behaviour [9,10,11,12]. The number of MCs in the brain can be sex-dependent, especially in young mice pups, where the total number of MCs in the preoptic area is nearly two times higher for males than females, potentially contributing to the gender bias in human neuropathology for diseases such as autism spectrum disorder (ASD) or schizophrenia [13,14]. MC numbers are also age-dependent, with MCs being most abundant in brains of individuals under 19 years old, a pattern probably related to the age involution [8,15,16].

P2X receptors (P2XRs) are membrane ligand-gated ion channels and are members of the purinergic receptor family [17,18]. Of the seven P2XR family members, only four of them (P2X1, P2X4, P2X6 and P2X7) have been shown to be expressed in MCs, with each of them playing an important role in regulating MC activities, such as Ca^+^ influx and degranulation [19,20,21]. P2XRs are also present in neurons and glial cells, where their engagement may affect the development of neuroinflammatory pathologies such as the Alzheimer’s disease (AD), Parkinson’s disease (PD) and Multiple sclerosis (MS) [22,23,24,25].

Here, we explore the effects of the P2XR-mediated MC activation on microglia, astrocytes and oligodendrocytes and its role in neuroinflammation.

## 2. MCs and Glial Cells in Neuroinflammation

While diseases like MS are well-known to be inflammatory in nature, it is now being increasingly recognised that many other neurological conditions, such as the degenerative diseases AD or PD, have an inflammatory component contributing to their debilitating pathology. There are many cell types that have now been implicated in neuroinflammation, including glial cells (such as astrocytes, microglia, and oligodendrocytes) and MCs [9,26]. 

Activation of these cells occurs in response to mediators released from surrounding immune cells and neurons undergoing necrosis or activation, such as complement, histamine, neurotransmitters (e.g., glutamate; gamma-aminobutyric acid), adenosine 5′-triphosphate (ATP), growth factors and cytokines (e.g., TNF-α, IFN-γ, IL-17). The subsequent release of inflammatory cytokines (e.g., TNF-α, IL-1β, IL-6, IL-10), reactive oxygen species and nitric oxide by activated glial cells greatly amplifies the inflammatory response and promotes neurotoxicity [27,28,29].

In terms of the glial sub-types, astrocytes play an important role in BBB maintenance and homeostasis of the CNS and have a critical role in maintaining neurological functions by regulating synapse formation and its preservation during disease [30]. They account for around 25% of the brain volume, making them the most abundant glial cell population [31].

Microglia are innate immune phagocytes that account for about 10% of all glial cells [32]. They regulate brain development and maintenance of neuronal networks. During injury, microglia contribute to eliminate dead cells, protein plague aggregates and microbes by phagocytosis [33,34]. 

Oligodendrocytes, which are key players in myelin production and remyelination processes, comprise 5–8% of the glial cell population. Furthermore, these cells are producers of neurotrophic factors and stabilisers of neuronal connectivity. Myelinating oligodendrocytes are under the pressure of a high metabolic demand, iron, and lipids. Thus, this renders them highly sensitive to oxidative stress, to excessive ATP and/or activation of glutamate receptors, and hypoxic or ischemic damage. Their loss in numbers or dysfunction are evident in CNS trauma, ischaemia, autoimmune and AD pathogenesis [35,36].

MCs in the central nervous system (CNS) act as a source of a wide variety of proinflammatory mediators [9,37]. Furthermore, they are first responders in brain injury through their rapid degranulation and close proximity to neurons and glial cells thus contributing to blood brain barrier (BBB) breakdown and to both the initiation and exacerbation of the inflammatory response [38,39]. MC mediators may also function at a distance, with granular remnants being observed up to 500 μm away from the cell of origin, via the paracrine release of granular components [40].

Even though the number of brain resident MCs is very low, many cell products, such as histamine, tryptase, chymase and TNF-α, have a significant impact on the permeability of the BBB and the functionality of adjacent neurons and glial cells, such as astrocytes, microglia, and oligodendrocytes, thus possibly contributing to the development and exacerbation of neuroinflammatory diseases [41,42,43].

The role of MCs in CNS homeostasis and neuroprotection is yet not fully understood. The MC-mediated release of serotonin in mice has been found to promote spatial learning and memory [44], histamine was shown to regulate sleep/wake and food-seeking behaviour [45], and MC protease-4 protected CNS from post-traumatic brain inflammation [46]. In the human system, MCs differentiated from CD34+ blood progenitors were shown to synthesize and release angiogenin [47], that is neuroprotective and promotes survival of motor neurons [48]. Hendriksen et al. [9] described several mediators involved in neurogenesis, such as IL-6, IL-1β, TNF-α, histamine and serotonin, of which MCs could be a key source. Furthermore, a recent study by Lenz et al. [13] described brain resident MCs as a novel source of sex-specific variability during mice development, as the MCs-secreted histamine stimulates microglia to release prostaglandin E2, affecting the masculinisation process.

Emerging evidence indicates that MCs influence the onset and progression of neuroinflammatory diseases. In MS patients and rat models, large congregations of MCs were found in CNS areas of inflammatory demyelination [49], associated with elevated tryptase levels in the cerebrospinal fluid, BBB breakdown and neutrophil recruitment [50]. In a mouse model of PD, MC proteases were shown to induce release of chemokine (C-C motif) ligand (CCL) 2 from astrocytes, microglia, and neurons [51]. As CCL2 is a known chemoattractant for neutrophils, monocytes, and macrophages [52], its release from activated glial cells and neurons may attract these immune cells to the site. In a different mouse model of PD, Hong et al. [53] demonstrated recruitment of MCs into the substantia nigra through CCL2 release by microglia and astrocytes. The recruited MCs were shown to express tissue transglutaminase 2 (TG2), which is associated with release of various inflammatory mediators, such as histamine, TNF-α and leukotrienes. The same study also observed increased levels of histamine, IL-6, TNF-α, leukotrienes and TG2 activity in sera of human PD patients compared to healthy controls. Moreover, elevated levels of CCL2, together with increased numbers and degranulation of MCs were observed in brain sections and serum from C57BL/6 mice that suffered a traumatic brain injury [54]. In amyotrophic lateral sclerosis (ASL), MCs by secreting IL-6 and IL-10 were suggested to be early players in disease pathogenesis [55]. Degranulating MCs were also found to infiltrate skeletal muscle and areas along the peripheral motor pathway in SOD1 rats, a transgenic animal model with a G93A mutation in the SOD1 gene, designed to mimic ASL. This infiltration decreased upon administration of masitinib, a c-Kit receptor inhibitor drug [56].

In cerebral spinal fluid from AD patients, higher concentrations of serum amyloid A (SSA) were observed compared to normal controls [57]. A study by Barbierato et al. [58] showed that TNF-α stimulation upregulates SSA1 expression in glial cells from Sprague-Dawley rat cerebral cortices. As SSA activates and is a chemoattractant for MCs, its elevated expression during AD [59,60] might attract MCs to β-amyloid peptide (Aβ) deposit sites and thus suggesting a glial-mediated activation of MCs. However, at this stage these are only speculations and further investigations are needed.

Of note, most of the data on the role of MCs in the brain was obtained using murine models [13,50,51,53,58,61], with only a small level of validation in human systems [47,53,57]. Therefore, while data are suggestive, the degree to which MCs communicate with glial cells, how they influence brain microenvironment and if they can act as main culprits in neuroinflammatory diseases, is not yet fully understood. 

## 3. Expression of P2XRs and the Role of ATP and P2XR Activation in Neuroinflammation

### 3.1. ATP Release during Inflammation and Brain Pathology

In the CNS, extracellular ATP acts as a fast-excitatory neurotransmitter and an important mediator for neuron-glial, glial-glial, and neuron-neuron communication [62,63]. In a healthy tissue, ATP is found extracellularly at negligible concentrations, with neurons and glial cells carrying millimolar concentrations of ATP intracellularly [64]. This is released by Panx1 and Connexin channels, through vesicular transport or through membrane stress/damage [65]. For example, during inflammation, necrotic cells set free up to hundreds of µmol/L of ATP [66]. Increased concentrations of ATP have been detected in brain pathologies upon trauma, ischemia, epilepsy, PD or MS [67,68,69].

### 3.2. Expression of P2XRs and The Role of ATP and P2XR Activation in Glial Cells

Extracellular ATP is the sole activator of all P2XR family members and undergoes a rapid enzymatic degradation into adenosine diphosphate (ADP) upon extracellular release, which is then further degraded into adenosine monophosphate (AMP) and adenosine [70,71]. P2XR engagement by ATP activates Na^+^ and Ca^2+^ influx, and K^+^ efflux, resulting in the plasma membrane depolarisation and reorganization, release of cytokines (such as IL-6, IL-8 and TNF-α) and caspase activation [72,73]. The seven P2XRs exhibit different affinities for ATP and exist as homomeric or heteromeric receptors, with homomeric P2X5 and P2X6 potentially not being functional in humans. For P2X6 in particular heteromerization appears to be necessary for a correct folding and assembly [74,75,76].

Together with the P2XRs, eight different P2YRs belong to the purinergic receptor family. P2YRs are G-protein coupling receptors expressed and functional on MCs and glial cells and involved in AD or epilepsy disease pathogenesis [77,78,79,80]. However, since the P2YRs are activated by multiple mediators, such as ATP, ADP, UTP, UDP and UDP-glucose [81] and since this review only examines the unique role of ATP in linking glial and MC activities, these receptors will not be discussed further.

### 3.3. P2XR Expression in Astrocytes

In astrocytes, P2X1 exists as a homomeric, or as a P2X1/P2X5 heteromeric receptor, the latter having unique properties compared to its homomeric counterpart. For example, the P2X1/P2X5 heteromeric receptor in astrocytes has a higher sensitivity to ATP with no desensitization response compared to the homomeric P2X1 [82,83]. However, P2X1/P2X5 expression is age-dependent, with a lack of heterodimers in 6 month old mice [22]. It is therefore unlikely that this receptor has an impact on astrocytes activities in adult mice.

P2X2 activation in astrocytes was found to regulate GABAergic transmission and ASD like behaviour in C57BL/6J mice carrying a knockout of the type 2 inositol 1,4,5-trisphosphate 6 receptors (IP3R2) gene, as mutations in this gene are associated with ASD [84]. Activation of the P2X2 also led to an increase in mRNA expression of leukaemia inhibitory factor (LIF), a cytokine inhibiting cell differentiation, in astrocytes isolated from neonatal C57BL/6J mice. Thus, this contributing to the efficacy of electroconvulsive therapy in psychiatric disorders [85]. 

Expression of P2X3 was reported in astrocytes in Sprague-Dawley rats [86] and in primary astrocytes cultures obtained from rats cerebral cortex [87], with receptor activation modulating craniofacial neuropathic pain [88].

Regarding P2X4, there is still limited evidence of its expression in astrocytes. This was demonstrated by RT-PCR and immunohistochemistry in rat cells [89,90]. However, studies performed in GFAP promoter-controlled EGFP-expressing (GFAP/EGFP) transgenic mice and in vitro using hippocampal slices from transgenic GFAP/EGFP mice and Wistar rats did not detect any P2X4-mediated ATP-induced current [82,91].

Rat cortical astrocytes [87] and human astrocytes isolated from foetal cortex express P2X5 [92]. However, knowledge about the receptor functionality is restricted to the P2X1/P2X5 heterodimers [80].

The presence of P2X6 in astrocytes remains controversial, as RT-PCR and western blotting of primary astrocytes from rats cerebral cortex didn’t show any expression [87], whilst P2X6 expression was detected using qPCR in human astrocytes from foetal cortex [92] and in astrocytes end-feet derived from Sprague-Dawley rats [93]. 

P2X7 activation was shown to attenuate LPS-induced release of TNF-α in primary cultures of rat cortical astrocytes [94]. In contrast, stimulation of P2X7 in mouse astrocyte cultures resulted in the secretion of various transmitter molecules, such as glutamate or GABA [95], and of a MAP kinase-controlled secretion of CCL2 in the Sprague-Dawley rat astrocytes [96]. In the hippocampus of C57BL/6J mice, P2X7 activation with extracellular ATP resulted in the release of neurotransmitters from astrocytes, leading to the stimulation of surrounding neurons [97]. In human foetal astrocyte cultures, regulation of P2X7 was induced by IL-1β [98] and its expression was observed in astrocytes from post-mortem brain tissues sections in AD patients [99]. In SOD1 mice astrocytes, P2X7 activation contributed to their toxicity towards motor neurons [100]. 

### 3.4. P2XR Expression in Microglia

In both mouse and human microglia, P2X4 and P2X7 are highly expressed [101], while evidence for the expression of P2X1, P2X2, P2X3 and P2X6 remains controversial. In this regard, microglia cultures obtained from Sprague-Dawley rats and BV-2 (immortalized murine microglia) cells showed P2X1 expression [102,103], whilst C57BL/6J and SOD1 mice microglia cells displayed very low or no expression [104,105]. Xiang & Burnstock [106] showed P2X1 expression in Wistar rats microglia only at late stages of embryonic development and until day 30 of postnatal development, suggesting that the expression of P2X1 in animal models might be species and age dependent. In human microglia, voltage-clamp electrophysiology performed after ATP stimulation in two donors showed no evidence of rapid desensitising inward current expected from P2X1 and P2X3 engagement [107]. RNA sequencing studies by Chiu et al. [108] and Solga et al. [109] in microglia from SOD1 and C57BL/6 mice, detected either none or extremely low expression levels of P2X1, P2X2, P2X3, P2X5 and P2X6, respectively. On the contrary, western blot analysis of N9 murine microglial cell line showed the presence of P2X1, P2X2, P2X3 and P2X6 [110]. Thus, discrepancies in P2X1, P2X2, P2X3 and P2X6 expression were found not only between species but also in similar murine systems.

P2X4 plays a major role in the regulation of neuronal and glial functions, as peripheral damage induces an upregulation of P2X4 in microglia and affects the inflammatory response [111]. It appears that P2X4 in rat cultured microglia is predominantly stored intracellularly while membrane expression is rapidly upregulated through C-C chemokine receptor type (CCR) 2-mediated activation upon CCL2 or CCL12 ligand binding [112]. Stimulation of P2X4 in mice microglia leads to maintained mechanical hypersensitivity after nerve injury, through the release of brain-derived neurotrophic factor [113], which is a crucial signalling mediator between microglia and neurons [114]. Deletion of P2X4 in P2X4^−/−^ KO mice resulted in the absence of mechanical hypersensitivity after peripheral nerve lesion [113]. In a mouse model of experimental autoimmune encephalomyelitis (EAE), P2X4 was shown to be a modulator of microglia polarization and its increase in expression to be a marker of the neuroinflammatory response [115]. Furthermore, P2X4 was also suggested to contribute to the activation and migration of Lewis rat microglia into the site of a formalin-induced injury [116].

Activation of the P2X7 in mice microglia results in the activation of the inflammasome, release of TNF-α, CCL2, IL-6, IL-1β, and IL-18, and increased cell death [117,118]. In healthy human donors, microglia isolated from the cortex expressed functional P2X7, but no release of IL-1β or IL-18 was observed upon LPS priming and subsequent ATP stimulation. The authors hypothesized that the cultured cells switched from a M1 inflammatory phenotype to an anti-inflammatory M2 phenotype in the presence of serum contained in the culture medium, therefore possibly shifting the nature of the microglia behaviour [107]. 

P2X7 activity in microglia has been linked to several neuroinflammatory diseases. In a mouse model of AD, upregulation of P2X7 expression was observed in microglia in proximity to Aβ peptide aggregates, and this expression was further elevated in the later stages of Aβ pathology. The same results were then observed in AD patients, suggesting an importance of P2X7 in AD pathology [23,99,119]. P2X7 activation in microglia has also been linked to MS, stress, depression, and PD in Sprague-Dawley rats, Wistar rats and C57BL/6J mice models [24,25,120,121]. Upregulation of P2X7 expression in microglia was observed in SOD1 mice [122] and receptor activation was found to modulate autophagic flux, a homeostatic mechanism involved in degradation of damaged organelles and protein aggregates, whose abnormalities were reported in ASL [123]. Inhibition of P2X7 using brilliant blue G showed prolonged survival in female SOD1 mice [124], while administration of JNJ-47965567 P2X7 inhibitor in the same model did not alter ALS progression [125].

### 3.5. P2XR Expression in Oligodendrocytes

Expression of P2X1, P2X2 and P2X3 was observed in oligodendrocytes progenitor cells isolated from postnatal 1 day Wistar rats [126] and in human stem cell-derived oligodendrocytes progenitor cells [127], while was absent in mouse mature and progenitor oligodendrocytes [128].

P2X4 expression in oligodendrocytes was confirmed by western blot analysis, qPCR and RNA sequencing in mice, rats and human progenitor oligodendrocytes [126,127,128]. However, Zabala et al. [115] were unable to link receptor expression to functionality in these cells. P2X5 and P2X6 were not found in oligodendrocytes lineage cells [129]. 

In contrast to other P2XRs, the functionality of P2X7 in oligodendrocytes has been demonstrated by Matute et al. [130] where a continuous activation of P2X7 led to the oligodendrocytes’ death, due to the P2X7-mediated Ca^2+^ toxicity. Furthermore, an increase in P2X7 expression in oligodendrocytes was found in samples from patients with MS, in a mouse model in post-episodes of status epilepticus and during epilepsy [131], and in rat model of ischemic damage [132].

Overall, P2XR activities in glial cells are yet unclear, with the exception of P2X7, which engagement initiates the release of mediators whose nature differs between species [101,133]. 

### 3.6. Expression of P2XRs and The Role of ATP and P2XR Activation in MCs

In MCs, expression of P2X1, P2X4 and P2X7 has been confirmed by RT-PCR analysis in LAD2 cells and human lung MCs, and by proteomics analysis in human and mouse primary connective tissue MCs [19,134]. P2X6 expression has also been observed in LAD2 and human lung MCs, however its functionality has not been demonstrated yet [19,135]. 

The MC homomeric P2X1 binds ATP with high affinity, with only 1 μM needed to activate P2X1 in LAD2 cells [19]. Study by Wareham & Seward [20] observed that the engagement of the P2X1 triggers a fast and transient calcium influx and a prolonged exposure to even low ATP concentrations may lead to its desensitisation. Even though it was concluded that P2X1 activation in LAD2 cells does not trigger degranulation, it was not investigated further if the activation might lead to a release of specific mediators. 

Like the P2X1, P2X4 activation leads to a calcium influx into MCs without inducing degranulation. However, P2X4 activation with an ATP concentration of less than 300 μM significantly increased degranulation mediated by high-affinity IgE receptor or by G-coupled prostaglandin EP3 receptor stimulation in bone marrow-derived MCs (BMMCs) from C57BL/6 mice [21,136]. P2X4 stimulation by ATP was also shown to enhance antigen-induced phosphorylation of Syk and PLCγ signalling pathways in mice BMMCs, independent of the P2X4-mediated calcium influx [137]. Inhibition of P2X4, by the potent and selective benzodiazepine derivative 5-BDBD, in human lung MCs diminished release of cysteinyl leukotrienes [135].

To date, P2X7 is the only P2XR demonstrated to induce MC degranulation. Activation of P2X7 triggers degranulation of meningeal MCs derived from C57BL/6 mice and human LAD2 cells [20,138], resulting in the immediate release of many pre-stored inflammatory mediators (such as histamine, tryptase, chymase, IL-6, IL-1β, and CCL2), with others, such as IL-5, CCL3 and eicosanoids, being newly synthesised over time [139,140]. Furthermore Shimokawa et al. [141] reported that P2X7 activation by ATP induced the secretion of IL-33 in mouse BMMCs.

In contrast to glial cells, P2X1, P2X4, P2X6 and P2X7 expression has been observed in vivo and in vitro in murine and human MCs, with P2X7 detected in brain resident MCs [19,134,138].

### 3.7. Expression of P2XRs: Public Gene Expression Databases

The availability of public datasets such as the iFANTOM and ImmGen consortiums now provide a useful tool to glean additional information on the expression of P2XRs in MCs and glial cells. Table 1 summarises the relative logarithmic expression (RLE) of P2XRs in human skin MCs samples [142], cerebellum, cortex astrocytes, and oligodendrocyte precursors [143] in comparison to other cell types (FANTOM5; www.fantom.gsc.riken.jp accessed on 30 July 2021). The ImmGen consortium (www.immgen.org accessed on 30 July 2021) displays murine microarray data [144], reporting organ specific and tissue-dependent P2XR expression in MCs (Table 2). P2X1, P2X4 and P2X7 are mostly expressed in the peritoneal cavity while P2X2, P2X3, P2X5 and P2X6 expression is constitutive and shared between organs and tissues. It should be noted, that P2XR expression is lower in MCs and glial cells compared to other cell types, except for P2X1, which exhibits the highest expression in skin MCs.

## 4. Interactions and Cross-Talk between MCs and Glial Cells in Neuroinflammation: The Role of ATP and P2XRs

In recent years, there has been increased interest in the interactions and cross-talk between MCs and glial cells. For example, MC derived histamine and ATP altered phagocytic activities of cultured microglia from Wistar rat cells [145], peripheral surgery in C57BL6/J mice induced MC degranulation which led to BBB breakdown and microglial activation [146]. Furthermore, the MC degranulator C48/80 dosed to the hypothalamus of Sprague-Dawley rats induced microglia activation, phosphorylation of mitogen-activated protein kinase (MAPK) and increased expression of H_1_R, H_4_R, protease-activated receptor-2 (PAR2) and toll-like receptor (TLR) 4 receptors [147]. Interactions between MCs and astrocytes through CD40/CD40L receptors, that caused the release of IL-1β, IL-6, TNF-α and CCL2 from astrocytes, were observed in co-cultures of human MCs (HMC-1 cells and U87 cells) and mouse (BALB/c) astrocytes [148]. In in vitro co-cultures, rat peritoneal MCs engaged with oligodendrocytes in a bidirectional cross-talk by adhering to them and releasing granule content thereby causing morphological changes and initiating apoptosis [149]. 

**Figure 1:**
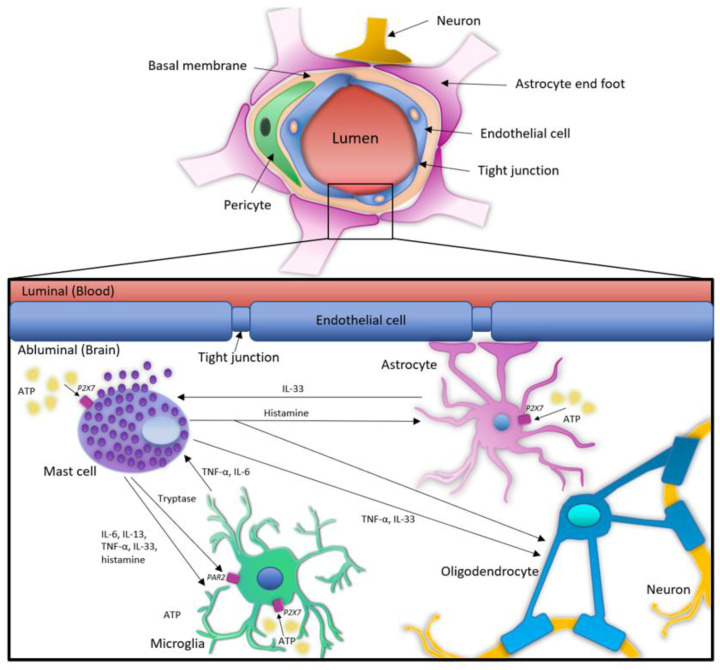
ATP-mediated MCs interactions with microglia, astrocytes, and oligodendrocytes. The blood brain barrier consists of a semi-permeable border composed of endothelial cells and tight junctions wrapped around a blood vessel. At the abluminal side of the blood brain barrier a variety of cell types can be found, such as resident glial cells or wandering immune cells. One of the immune cells found on the abluminal side are MCs that possess an ability to influence the function of microglia, astrocytes, and oligodendrocytes, through activation of P2XRs, resulting in a release of various mediators, such as of histamine, tryptase, IL-6, IL-13, TNF-α and IL-33. On the other hand, microglia can release TNF-α, IL-6 and potentially IL-33 upon P2XR activation, which will result in altered function of mast cells and a stronger immune response.

At present, despite limited direct evidence of how activation of P2XRs in MCs affects glial cells and vice versa, from indirect evidence we can speculate the likely effect that activation of P2XRs could have on the communication between MCs and glial cells (Figure 1 and Table 3). 

Upon P2XR activation, MCs release a wide range of inflammatory mediators, that modulate the activity of cells in their proximity [150,151,152]. For example, MC tryptase activates microglial PAR2, resulting in the release of pro-inflammatory mediators such as TNF-α and IL-6, which will in turn upregulate PAR2 expression on microglia, cause apoptosis in oligodendrocytes and release of glutamate from astrocytes [153,154,155,156]. Tryptase also plays a role in promoting the expression of P2X4 on microglia, where the activation of this receptor leads to a release of brain-derived neurotrophic factor [157]. On the other hand, activation of microglia through P2X7 and the release of IL-6 and TNF-α, could affect MC secretion of IL-13, IL-4, and upregulation of TLR2/TLR4 receptors [9,158].

Activation of P2X7 in MCs also triggers the release of histamine. About 50% of all histamine present in the brain is released by MCs [159] and stimulates microglia to secrete TNF-α, IL-1β and IL-6, while suppressing production of TNF-α and IL-1β in astrocytes [9,160,161,162]. Histamine was also shown to negatively regulate the differentiation of oligodendrocytes through H2 receptor engagement [163], while inhibition of H3 receptor promoted murine oligodendrocytes differentiation and remyelination [164].

MCs secrete IL-33 upon P2X7 activation [165], which in mouse models promotes microglia migration to sites of CNS injury [166] and the release of pro-inflammatory mediators that activate endothelial cells (thus facilitating leukocyte recruitment) [167], but inhibits myelination [168]. ATP-mediated release of IL-33 in microglia has been suggested [169] but remains controversial. IL-33 expression in microglia has been demonstrated in brain samples of MS patients and in wild type C57BL/6J murine brain [168,170]. However, primary cultures of C57BL/6J mouse microglia did not show any detectable levels of IL-33 [171,172]. In contrast, astrocyte-derived IL-33 was found to delay disease onset in the ASL transgenic mice model and promote microglia synapse engulfment in C57BL/6J mice [173,174]. IL-33 is known as an alarmin and is a potent modulator of MC activities and contributor to allergic inflammation. In MCs, IL-33 promotes the release of soluble ST2 receptor and enhances MC adhesion to laminin and fibronectin [175]. However, whether specific MC activities are regulated by glial cell-produced IL-33 is yet to be determined.

While there is only limited evidence, especially in the human system, on how P2XR engagement affects communication between MCs and glial cells, the data are suggestive that P2XR activation could be a key mechanism in regulating cell-cell interactions in the brain. 

**Table 3:** MCs and glial cells activities induced by P2XR engagement.

	P2XR Induced Activities		
Mediators	Glial Cells	MCs	References
Tryptase	Upregulation of P2X4 on microglia	N/A	[157]
PAR2	Release of MC tryptase activates PAR2 receptor on microglia	Activation of PAR2 receptor in microglia results in TNF and IL-6 release, affecting MCs	[154,155,156]
TNF-α/IL-6	Apoptosis in oligodendrocytes; glutamate release from astrocytes	Secretion of IL-13 and IL-4 from MCs, together with upregulation of TLR receptors	[9,153,158]
Histamine	Release of TNF-α, IL-1β and IL-6 from microglia; inhibition of TNF-α and IL-1β expression in astrocytes; negative regulation of oligodendrocytes differentiation	N/A	[160,161,162,163,164]
IL-33	Promotion of microglia migration to site of CNS injury and release of pro-inflammatory mediators; inhibition of myelination by oligodendrocytes; release of IL-33 from astrocytes delays ASL disease onset and promotes microglia synapse engulfment	Functions as alarmin on MCs, affecting activation status and mediator release	[166,167,168,173,174,175]

## 5. Concluding Remarks

MCs, microglia, astrocytes, and oligodendrocytes play important roles in response to the release of ATP during neuroinflammation. However, overall, it appears that P2X7 expressed in MCs might have the most influential effect on onset and progression of neuroinflammatory diseases, as its activation results in MC degranulation and the release of pro-inflammatory cytokines that have a significant downstream impact on microglia, astrocytes, and oligodendrocytes activities. Whether this regulation is bi-directional and ATP-mediated activation of P2XRs on microglia, astrocytes, and oligodendrocytes direct MC activities remains unclear. More remains to be eluded, but it is possible that in future the therapeutic blockade of MC P2X7 in AD, PD, or MS, perhaps with brain permeable P2X7 antagonists, could ameliorate the downstream pathological effect on glial cells and prove beneficial for patients with neuroinflammatory diseases.

## Figures and Tables

**Table 1:** Relative logarithmic expression (RLE) of P2XRs in MCs, astrocytes, and oligodendrocytes. P2XR RLE expression in skin MCs [142], cerebellum and cortex astrocyte and oligodendrocyte precursors [143] was obtained using Fantom 5 and compared to cell types with the highest RLE for a given receptor.

Gene	MCs	Astrocytes		Oligodendrocytes	Cell Types/Tissues with Highest RLE
	Skin (*n* = 4)	Cerebellum (*n* = 3)	Cortex (*n* = 3)	Precursors (*n* = 1)	
P2X1	337.186	3.017	3.345	1.692	337.186 (MCs)
P2X2	0.032	0	0	0	15.242 (Seminal vesicle)
P2X3	25.120	67.947	96.481	51.310	282.548 (Smooth muscle cells)
P2X4	27.792	6.232	4.140	6.202	679.838(CD14+ monocytes)
P2X5	71.924	363.640	0.814	294.329	2237.838 (Bronchial epithelial cells)
P2X6	2.373	1.187	65.063	0	25.101(Cerebellum)
P2X7	14.628	0.164	0.324	0.564	692.939 (CD14+ monocytes)

**Table 2:** P2XR expression in mouse MCs of different tissue origin. Shown are robust multichip average (RMA) normalized values. Data were obtained from the ImmGen consortium [144].

Gene	MC Origin				
	Skin (*n* = 3)	Peritoneal Cavity (*n* = 3)	Tongue (*n* = 3)	Oesophagus (*n* = 3)	Trachea (*n* = 3)
P2X1	1356.95	2106.25	1715.59	1105.08	1535.27
P2X2	77.3146	73.6825	87.4627	85.148	76.2191
P2X3	65.3869	64.7405	61.4027	49.9133	51.1301
P2X4	1289.72	3261.39	1656.1	1986.01	2416.66
P2X5	165.822	117.41	146.774	142.33	143.221
P2X6	112.974	97.5069	107.431	122.073	100.487
P2X7	299.952	2413.83	695.205	871.043	1146.44

## Abbreviations

AD	Alzheimer’s disease
Aβ	β-amyloid peptide
ADP	Adenosine diphosphate
AMP	Adenosine monophosphate
ALS	Amyotrophic lateral sclerosis
ASD	Autism spectrum disorder
ATP	Adenosine 5′-triphosphate
BBB	Blood brain barrier
BMMCs	Bone marrow-derived mast cells
CCL	Chemokine (C-C motif) ligand
CCR	C-C chemokine receptor type
CNS	Central nervous system
EAE	Experimental autoimmune encephalomyelitis
FcɛRI	High-affinity IgE receptor
FcγR	Fc-gamma receptor
IL	Interleukin
KO	Knockout
LPS	Lipopolysaccharide
MAPK	Mitogen-activated protein kinase
MC	Mast cell
MS	Multiple sclerosis
PAR2	Protease activated receptor 2
PD	Parkinson’s disease
SSA	Serum amyloid A
TG2	Tissue transglutaminase 2
TLR	Toll-like receptor
TNF-α	Tumor necrosis factor alpha
